# Editorial: Uncovering the neural correlates of prism adaptation: Evidence from the brain network approach

**DOI:** 10.3389/fpsyg.2022.1076307

**Published:** 2022-11-15

**Authors:** Francesco Panico, Selene Schintu, Luigi Trojano

**Affiliations:** ^1^Department of Psychology, University of Campania ‘Luigi Vanvitelli', Caserta, Italy; ^2^CIMeC—Center for Mind/Brain Sciences, University of Trento, Trento, Italy; ^3^Behavioral Neurology Unit, National Institute of Neurological Disorders and Stroke, Bethesda, MD, United States; ^4^Department of Psychological and Brain Sciences, The George Washington University, Washington, DC, United States

**Keywords:** prism adaptation, sensorimotor plasticity, spatial attention, cognition, brain networks, dynamic connectivity, neglect

Since early applications of Prism Adaptation (PA) in the rehabilitation of unilateral spatial neglect (Rossetti et al., 1998; Frassinetti et al., 2002) a plethora of studies on brain-damaged and healthy individuals revealed that PA can exert *supramodal* effects on high-order cognition, such as spatial attention, time perception, auditory perception, and reward-based learning (e.g., Schintu et al., 2018; Anelli and Frassinetti, 2019; Michel et al., 2019). These results prompted further research aimed at investigating the neural correlates of PA-induced sensorimotor and cognitive aftereffects (Panico et al., 2020, 2021; Schintu et al., 2020, 2022), and understanding their temporal dynamics. The role of different variables, such as the state of the system at baseline, the hand used during adaptation and the direction of the prismatic displacement (Schintu et al., 2017; McIntosh et al., 2019) has been largely investigated, but there are still open questions about the processes of generalization, transfer, expansion, and consolidation taking place during and after PA (Prablanc et al., 2020).

PA modulates cognition according to the direction of the visual displacement, which surprisingly interacts with the system (intact vs. damaged) that is being adapted. It is well-known that right-deviating PA can ameliorate neglect symptoms, whereas left PA is ineffective. However, left PA produces relevant cognitive changes in healthy individuals, whereas right PA does not. The review paper by Clarke et al. tackled this long-standing conundrum and proposed a model accounting for such asymmetry in PA-induced effects. The model posits that PA induces instability in the synaptic organization in the posterior parietal cortex (PPC) contralateral to the optical deviation, consequently affecting the ventral attentional network (VAN). According to the model, right PA may produce its beneficial effects in right hemisphere stroke patients with neglect by reshuffling the left PPC representations, thus compensating for the functions of the damaged right VAN. Differently, left PA would exert no effect in these patients since the reshuffling would occur in the lesioned right PPC. In healthy individuals, right PA would not produce any effect as healthy individuals may continue to rely on their right (intact) VAN, not taking advantage of the reshuffled left PPC. In contrast, left PA would determine a reshuffling in the right PPC, thus accounting for neglect-like behavior.

Another long-standing question in this field is *how* sensorimotor adaptation to laterally displaced vision can prompt changes in cognition. In their fMRI study, Schintu et al. used structural equation modeling (SEM), a form of effective connectivity analysis, to uncover connectivity directionality and causality among the network underpinning PA (Schintu et al., 2020). These findings provided the first direct evidence that PA is underpinned by two functionally distinct subnetworks, a parietal–temporal network responsible for sensorimotor aftereffects and a fronto-cerebellar network responsible for cognitive aftereffects, consistent with the model recently put forward by Panico et al. (2020) positing a distinction between networks responsible for PA sensorimotor and cognitive aftereffects.

While the network underlying PA is still object of discussion, there is general agreement on the core role of the cerebellum during the different stages of the sensorimotor adaptation: from error correction to aftereffect development. Fleury et al. using anodal transcranial Direct Current Stimulation assessed the role of the cerebellum in the transfer of prism aftereffects from a throwing task to a pointing task as quantified by behavioral and kinematic measures (Fleury et al., 2021). Although the overall magnitude of transfer was not modified by the stimulation, the results showed that facilitatory stimulation affected the evolution of pointing errors during transfer assessment. Moreover, the inspection of kinematics parameters during transfer assessment suggested a lasting trace of adaptation related to transfer.

Concerning the variables affecting PA-induced aftereffect, in a fMRI study on healthy participants Farron et al. assessed whether the hand (right vs. left) used during right PA affected the process of visuomotor adaptation and, therefore, the reshaping of the attentional system. The authors compared brain activation patterns to visual targets in left, central, and right visual field before and after PA. They observed that left and central targets enhanced activation in the left PPC following right PA, and that this effect was modulated by the hand used during adaptation. This study clearly showed that the hand used during adaptation can affect the degree to which right PA modulates the functioning of the VAN. By using a behavioral approach, Bonnet et al. instead explored the effects induced by vertical PA at both sensorimotor and cognitive levels. Bonnet et al.'s experiments showed that, unlike lateral PA, the aftereffects of vertical PA also occurred in the auditory domain, but not in vertical visuospatial representations. These findings suggested that both vertical and lateral PA share a common substrate in the auditory modality, and that vertical PA does not act on the neural substrate of vertical visuospatial representations.

Whether PA could be implemented in a virtual reality environment and how sensory parameters (verbal vs. visual) could influence PA-induced aftereffect was investigated by Bourgeois et al. The authors compared the effect of visual vs. auditory-verbal feedback during the adaptation phase on visuospatial judgments implementing a simulated prism exposure in immersed virtual reality. Results confirmed that a virtual PA procedure can induce PA aftereffects, and showed that only the visual, but not the auditory-verbal feedback produces aftereffects following adaptation to virtual prisms, pointing out that the effects of sensorimotor adaptation operate in a visually aligned coordinate system.

The goal of this Research Topic was to provide evidence on the PA neural correlates and on its mechanisms of action from a network approach. According to this approach, the adaptive processes of sensorimotor adaptation to prims and their consequent expansion to cognition arise from the tight interaction between functionally interconnected brain regions. Taken together the studies presented in this Research Topic confirmed from different perspectives that PA is a deceptively simple technique. The PA-induced sensorimotor and cognitive effects are subtended by the dynamic interplay of likely distinct frontal-parietal-cerebellar networks, whose activation can be modulated by several variables related to the adaptation procedures.

## Author contributions

All authors listed have made a substantial, direct, and intellectual contribution to the work and approved it for publication.

## Funding

FP was funded by PON Green/Innovation 2014-2020 (DM 1062, 10/08/2021).

## Conflict of interest

The authors declare that the research was conducted in the absence of any commercial or financial relationships that could be construed as a potential conflict of interest.

## Publisher's note

All claims expressed in this article are solely those of the authors and do not necessarily represent those of their affiliated organizations, or those of the publisher, the editors and the reviewers. Any product that may be evaluated in this article, or claim that may be made by its manufacturer, is not guaranteed or endorsed by the publisher.

## References

[B1] Anelli F. Frassinetti F. (2019). Prisms for timing better: a review on application of prism adaptation on temporal domain. Cortex 119, 583–593. 10.1016/j.cortex.2018.10.017 30503631

[B2] Fleury L. Panico F. Foncelle A. Revol P. Delporte L. Jacquin-Courtois S.. (2021). Non-invasive brain stimulation shows possible cerebellar contribution in transfer of prism adaptation after-effects from pointing to throwing movements. Brain Cogn. 151, 105735. 10.1016/j.bandc.2021.105735 33945939

[B3] Frassinetti F. Angeli V. Meneghello F. Avanzi S. Làdavas E. (2002). Long-lasting amelioration of visuospatial neglect by prism adaptation. Brain 125, 608–623. 10.1093/brain/awf056 11872617

[B4] McIntosh R. D. Brown B. M. A. Young L. (2019). Meta-analysis of the visuospatial aftereffects of prism adaptation, with two novel experiments. Cortex 111, 256–273. 10.1016/j.cortex.2018.11.013 30530268

[B5] Michel C. Bonnet C. Podor B. Bard P. Poulin-Charronnat B. (2019). Wearing prisms to hear differently: after-effects of prism adaptation on auditory perception. Cortex 115, 123–132. 10.1016/j.cortex.2019.01.015 30822612

[B6] Panico F. Fleury L. Trojano L. Rossetti Y. (2021). Prism adaptation in M1. J. Cogn. Neurosci. 33, 563–573. 10.1162/jocn_a_01668 33378244

[B7] Panico F. Rossetti Y. Trojano L. (2020). On the mechanisms underlying Prism Adaptation: a review of neuro-imaging and neuro-stimulation studies. Cortex 123, 57–71. 10.1016/j.cortex.2019.10.003 31759324

[B8] Prablanc C. Panico F. Fleury L. Pisella L. Nijboer T. Kitazawa S.. (2020). Adapting terminology: clarifying prism adaptation vocabulary, concepts, and methods. Neurosci. Res. 153, 8–21. 10.1016/j.neures.2019.03.003 30910735

[B9] Rossetti Y. Rode G. Pisella L. Farné A. Li L. Boisson D.. (1998). Prism adaptation to a rightward optical deviation rehabilitates left hemispatial neglect. Nature 395, 166–169. 10.1038/25988 9744273

[B10] Schintu S. Freedberg M. Alam Z. M. Shomstein S. Wassermann E. M. (2018). Left-shifting prism adaptation boosts reward-based learning. Cortex 109, 279–286. 10.1016/j.cortex.2018.09.021 30399479 PMC7327780

[B11] Schintu S. Freedberg M. Gotts S. J. Cunningham C. A. Alam Z. M. Shomstein S.. (2020). Prism adaptation modulates connectivity of the intraparietal sulcus with multiple brain networks. Cerebr. Cortex 30, 4747. 10.1093/cercor/bhaa032 32313949 PMC7526755

[B12] Schintu S. Kravitz D. J. Silson E. H. Cunningham C. A. Wassermann E. M. Shomstein S. (2022). Dynamic changes in spatial representation within the posterior parietal cortex in response to visuomotor adaptation. Cerebr. Cortex 2022, bhac298. 10.1093/cercor/bhac298 35989306 PMC10068280

[B13] Schintu S. Patané I. Caldano M. Salemme R. Reilly K. T. Pisella L.. (2017). The asymmetrical effect of leftward and rightward prisms on intact visuospatial cognition. Cortex 97, 23–31. 10.1016/j.cortex.2017.09.015 29078083 PMC5716840

